# How to make healthy early childhood development more likely in marginalized Roma communities: a concept mapping approach

**DOI:** 10.1186/s12939-022-01637-0

**Published:** 2022-03-27

**Authors:** Shoshana Chovan, Daniela Filakovska Bobakova, Lucia Bosakova, Andrea Madarasova Geckova, Sijmen A. Reijneveld, Marlou L. A. de Kroon

**Affiliations:** 1 Graduate School Kosice Institute for Society and Health, PJ Safarik University, Trieda SNP 1, 040 01 Kosice, Slovak Republic; 2 Department of Health Psychology and Methodology of Research, Medical Faculty, PJ Safarik University, Trieda SNP 1, 040 01 Kosice, Slovak Republic; 3 Olomouc University Social Health Institute, Palacky University in Olomouc, Univerzitni 22, 771 11 Olomouc, Czech Republic; 4 Department of Community & Occupational Medicine, University Medical Center Groningen, University of Groningen, Antonius Deusinglaan 1, 9713 AV Groningen, the Netherlands; 5 Environment and Health, Youth Health Care, University of Leuven, KU Leuven, Kapucijnenvoer 35, 3000 Leuven, Belgium

**Keywords:** Early childhood development, Marginalized Roma communities, Inequalities

## Abstract

**Background:**

This study aims to assess which measures could improve the healthy early childhood development of children from marginalized Roma communities and to identify priority measures.

**Methods:**

Concept mapping approach was used, using mixed methods. In total 54 professionals, including social workers, educators, health care providers, municipality representatives, and project managers participated in our study.

**Results:**

Four distinct clusters of measures targeting living conditions, public resources, healthcare and community interventions, and 27 individual priority measures of highest urgency and feasibility were identified. The cluster ‘Targeting living conditions’, was rated as the most urgent but least feasible, whereas the cluster ‘Targeting health care’, was considered least urgent but most feasible. Among the 27 priority measures, ‘Planning parenthood’ and ‘Scaling up existing projects’ had the highest priority.

**Conclusion:**

Our results reflect the public and political discourse and indicate significant barriers to implementation. Reducing inequalities in early childhood needs to be addressed through coordinated efforts.

## Introduction

The period from conception to the age of 3 years is characterized by the most rapid growth of mental and socioemotional capacities [47], mirroring the rapid development of brain architecture [39]. Healthy child development is an outcome of a continuous ongoing process, where the needs of the child are met, and the child acquires the skills and abilities needed to reach his or her full potential later in life [49]. Protective factors and interventions early in the developmental course have the greatest positive impact and are the most cost-effective compared to those later in life [48]. Additionally, it has been shown that the largest financial returns and the greatest effects are generated by policies focusing on delivering effective interventions to the most vulnerable populations [48].

In Slovakia, Roma living in the marginalized communities make up one of the most vulnerable groups threatened by multiple forms of disadvantage. “Marginalized communities are separated or segregated communities, excluded from mainstream social, economic, educational and cultural life [44]. The separated type refers to a Roma population concentrated in a certain part of a town or village – either inside or on the outskirts; the segregated type refers to a settlement that is remote from towns and villages or separated from them by a physical barrier [17].

Roma are the largest ethnic minority in Europe [9]. “Approximately 440 thousand Roma are living in Slovakia, of whom 53.5% live in separated or segregated communities. The rest lives scattered among the majority population [35].” People living in marginalized Roma communities are most often disadvantaged by lower education, unemployment, receiving social benefits, struggling with bills and lacking standard household facilities (sewage system, water supply, flush toilet, bathroom or shower, electricity) [31]. Such contextual characteristics can endanger healthy development in early childhood [13, 52].

The limit available evidence shows that the unfavourable conditions in early childhood are reflected in the poorer health status of Roma children [40]. This evidence shows that disparities in health begin early, with perinatal and infant mortality rates significantly higher in districts with a higher proportion of the population living in marginalized Roma communities [41]. Additionally, Roma children constitute 24.2% of all cases of sudden infant death syndrome in Slovakia [28], i.e., much more than the estimated share of Roma children in the general population, i.e., 13.3% [54]. Moreover, Roma children suffer more often from infectious diseases, injuries, poisoning, burns, respiratory diseases and chronic diseases than other children [2].

In addition to the above-mentioned contextual characteristics of the environment, poor access to health care also contributes to the poorer health status of Roma children. Barriers in access to health care, such as lack of funds for travel costs or pharmaceuticals, bad travel connections, bad previous experiences, fear or distrust [25], result in 36% lower health care use among marginalized Roma compared to the general population [34]. Therefore, Roma not only have poorer health but also use the health care system to a lesser extent, thus increasing their health disadvantage. They experience similar barriers to other health care services and to education, including early diagnosis and intervention [19, 34, 53]. In addition, Roma children younger than 3 years of age from these marginalized communities have poor access to early childhood health and educational programmes. Only a few early childhood education programmes are available for Roma, available only in a few Roma communities, and these are almost exclusively offered by non-governmental organizations [37].

The differences in health between Roma and non-Roma are well-documented, but evidence is still lacking on how to increase the chances for healthy early development of children in marginalized Roma communities [19, 53]. Therefore, the aim of our study was to assess the perceptions of professionals from different fields working with marginalized Roma communities on potential measures (i.e. interventions and policy changes) designed to improve the healthy early childhood development of children living in marginalized Roma communities and to identify priority measures on the basis of urgency and feasibility, as judged by these professionals.

## Methods

### Design

We used a group concept mapping (GCM) approach to structure the interventions aimed at increasing the chances for healthy early childhood development, as proposed by professionals who in their praxis are directly or indirectly involved with Roma living in marginalized communities. Group concept mapping is a participative research method comprising a mix of qualitative data collection and interpretation and quantitative data analysis [26].

The design of our study was informed by the theoretical Biodevelopmental framework for understanding the origins of disparities in learning, behaviour and health [46]. Within this framework, the foundations of healthy development and sources of early adversity are described on several levels i.e. the environment of relationships, the physical, chemical and built environments, and nutrition. This framework was elaborated by Shonkoff [46] to inform policy targeted at young children, particularly the most vulnerable ones. Based on this framework, we aimed to recruit professionals from different backgrounds with a deep understanding of the various determinants influencing healthy development and the sources of early adversities. (See Sample below.) In addition, we used the Biodevelopmental framework for structuring the data and interpreting the results.

### Sample

Purposive sampling techniques were used to recruit professionals working with marginalized Roma communities from Slovakia from both public and non-governmental sectors of different levels of work hierarchy across these categories: health care providers, social workers, community centre workers, early childhood educators, special educators, health mediators, experts in early childhood development and policymakers. Since the expert field of early childhood education and care is relatively small in Slovakia, we approached professionals with whom we had previously built rapport or professionals recommended by other professionals. We initially addressed 79 professionals, 54 of whom agreed to take part in the study (response rate of 69.2%).

The final sample for the brainstorming step consisted of 54 participants and for the sorting/rating step 40 participants for the GCM procedure, see Procedure below. “We lost some participants between the brainstorming and sorting/rating due to the fact that some of them decided not to partake in the next steps because of their work overload. This aligns with the GCM methodology, which takes into account some losses of participants between the brainstorming and sorting-rating step without bias being likely [24]. The sample size in each step of this study was sufficient to meet the statistical requirements for obtaining valid and reliable results [24].

### Procedure

**Fig. 1:**
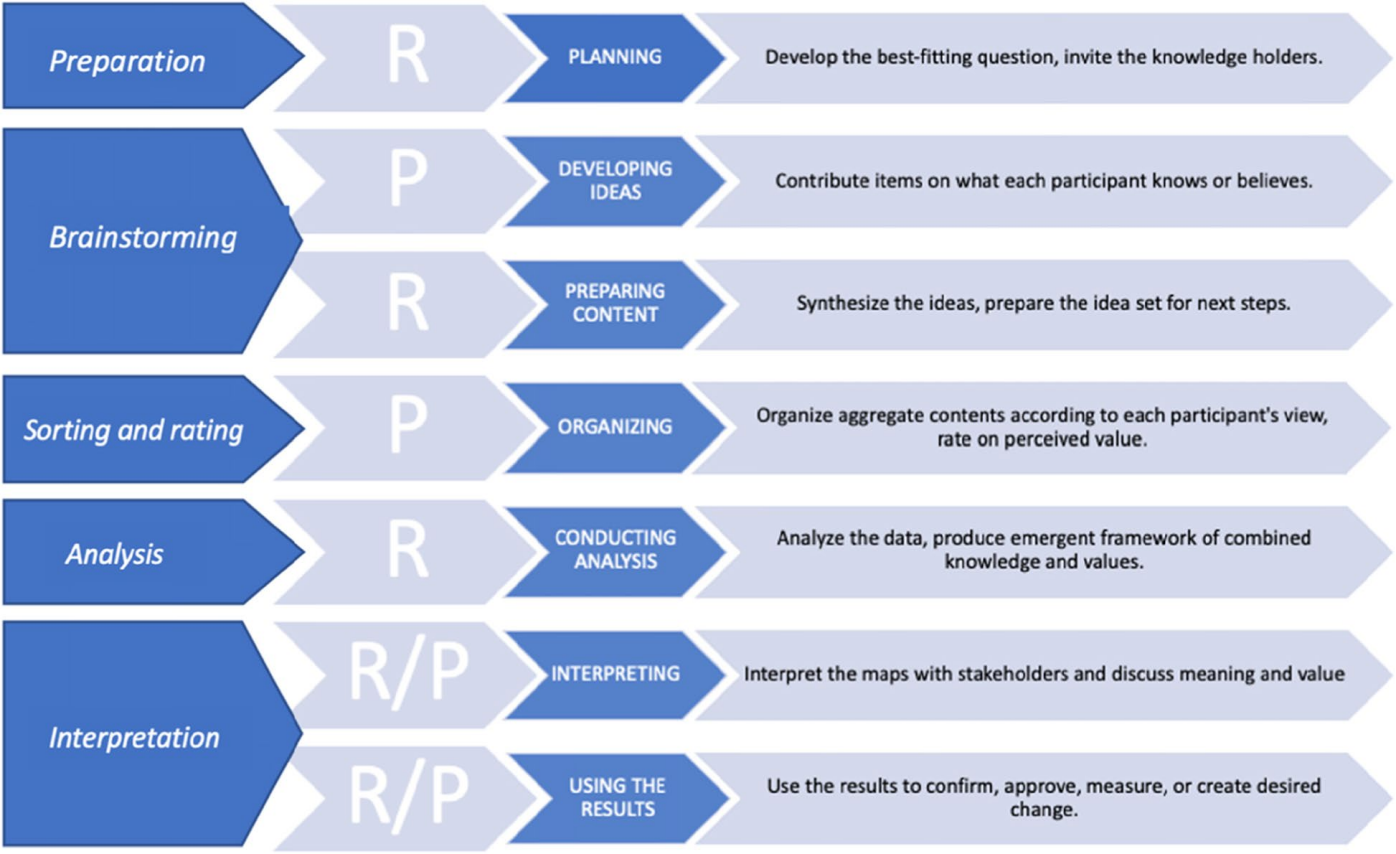
The Basic GCM Process (responsibility of P = participants, R = researchers) ([26], adapted)

We applied the general GCM procedure consisting of five steps: preparation, brainstorming, sorting and rating, analysis, and interpretation [27, 43]. Figure 1 illustrates this process and the responsibilities of both the researchers and the participants.

In the first step, the *preparation phase*, we formulated the research question, also known as the focus prompt: “What needs to be done to make the chances for healthy early childhood development of children from marginalized Roma communities equal with the chances of children from the majority population? The following explanations were given to the participants in order to make sure, that the focus prompt is clear to all participants: “By ‘early childhood’ we mean the period from conception to 3 years of age. By ‘healthy development’ we mean development that enables a child to reach his or her full potential in later life. This means healthy, age-appropriate growth and development of cognitive, social, emotional, language and motor skills. By ‘making chances equal’ we mean enhancing the starting positions similarly, which we know is worse compared to majority population. Roma children are disadvantaged when entering school and in their further life.”

The potential participants were identified in line with the Biodevelopmental framework [46] and contacted, and the schedule of the project was set. We decided to conduct the study online, using conference calls and the groupwisdom™ platform (https://groupwisdom.com/, a platform where each step of the GCM can be performed online). Prior to the data collection, we organized a conference call attended by the research team and the participants, where the aim of the study and the GCM procedure was explained and discussed.

In the second step, we organized the *brainstorming* phase using the online groupwisdom™ platform. Participants were asked to sign the consent form first and then to answer the focus prompt. In order to protect the participants from potential power-relations interplaying during the brainstorming part of the study, this phase was made anonymous. Due to the anonymous nature of this step, no demographic data was collected at that moment. This is in line with the GCM methodology, as these questions are most frequently asked during the sorting and rating phase. Participants were encouraged to generate as many statements as needed. To prepare a master list (a final list of statements) for the subsequent sorting-rating phase, we performed a synthesis of the statements and a qualitative review. We merged semantically similar statements and split those which contained more than a single concept with the aim of removing redundant and overlapping concepts and creating a reduced, concise set of statements. We further removed statements according to these criteria: statements not answering the focal question, statements not possible to handle by policy (e.g. containing rather personal issues such as family support) and statements describing a problem rather than a solution. The master list containing the final set of items was once again sent to the participants for commenting and clarification.

In the third step, the *sorting-rating* phase, we asked the participants to *sort* the statements into groups or piles of similarly themed statements and to create a descriptive label for each group based on what they view as the unifying topic or content of each group. We then asked them to *rate* these statements according to two selected domains of interest, i.e., urgency and feasibility, on a 7-point Likert scale (1 – not urgent/not feasible, 7 – very urgent/very feasible). Sorting and rating were performed using the groupwisdom™ platform.

In the fourth step, the *analytic phase*, we made the final data categorization. A quality review was performed to check if all participants followed the sorting and rating guidelines (rated at least 75% of the task or did not give negligent answers) and analysed the data using the groupwisdom™ platform. The findings of the analyses were discussed within the research team, and the resulting 4-cluster solution was chosen based on the most consistent cluster map. Cluster labels were discussed and decided on in an expert group consisting of 6 researchers, and subtopics were identified in each of the clusters.

In the *interpretation phase*, the final cluster solution and cluster labels proposed by the expert group were sent to the participants to gain their feedback on these results and their interpretation of the resulting maps.

### Statistical analysis and reporting

As data handling and data analysis occur simultaneously in GCM, this paragraph discusses them both in their sequence as applied, with some initial data handling already presented in the previously described steps 2 and 4. First, we performed a descriptive analysis to describe the background characteristic of our sorting-rating sample.

Second, we categorized using cluster analysis the perceptions of professionals from different fields working with marginalized Roma communities regarding potential measures to improve the healthy early childhood development of Roma children. In that analysis we clustered the generated statements into a cluster point map using a multidimensional scaling and hierarchical cluster analysis [27]. In this map, a point represents a single statement, the distance between the points indicates how often participants sorted particular statements into the same group, and the size of the cluster indicates the degree to which its various contributing statements are related. The expert group computed the stress index for a varying number of clusters (3–13, i.e. the highest and the lowest desired number of clusters, as sorted by participants) and used bridging/anchoring analysis and spanning analysis. The first analysis shows the relationship of the statement to its location on the map; the latter one visualises the statement’s strength of connection to every other item on the map [26]. The expert group finally proposed a 4-cluster solution.

Third, to identify priority clusters according to urgency and feasibility, we generated cluster rating maps, where more layers indicate more urgency and feasibility, respectively. To identify separate priority measures, we produced a Go-Zone map, i.e., an X–Y graph which compares items across two rating criteria and is divided into quadrants above and below the mean value of each rating variable [8]. We checked the model fit using the stress-index i.e., the degree to which the distances on the map are discrepant from the values in the input similarity matrix; a high stress-index value indicates a greater discrepancy (i.e., the map does not represent the input data well) [27, 29]. Within a GCM context, stress-index values should range from 0.10 to 0.35, with lower values indicating a better fit [20]. All analyses were performed using the groupwisdom™ software (https://groupwisdom.com/).

## Results

### Background characteristics of the sample

The sorting-rating sample, consisted of 31 women and 9 men, with a mean age of 42.5 years (minimum = 27, maximum = 59; years of age were recorded in rounded numbers). Out of all the participants, 33 had completed university education (82.5%), 6 had completed secondary education (15.0%) and 1 had completed elementary education (2.5%). Direct contact with marginalized Roma in their work was reported by 34 participants (85.0%); 6 participants (15.0%) did not have direct contact with marginalized Roma in their work. These 6 participants were from higher levels of the work hierarchy and despite not working directly with the Roma, their expertise was relevant to the research question (e.g. they focus on policy making targeted at inclusive policies). The mean period spent in the current work position was 11.2 years (minimum = 0.5 years, maximum = 30.0 years). Participants who have worked in their current position only for a short time had previous experience working in related fields.

### Final cluster solution of proposed measures

Participants generated 178 proposals for measures in the brainstorming phase. After review and synthesis of the statements, we created a master list containing a final set of 90 proposed measures. Participants sorted the 90 proposed measures into 3 to 13 groups. The expert group consisting of 6 researchers chose and approved the final 4-cluster solution. The expert group agreed upon the final cluster labels and topics identified within each cluster, which were as follows.

Cluster 1 represents *Public resources for instrumental support* and contains two topics, *Financial and institutional frameworks* and *Tools for instrumental support.* This cluster contained measures reducing barriers in access to services, ensuring funding to programmes targeting children living in marginalized Roma communities, and providing basic equipment and adequate nutrition. An example of a measure from Cluster one is “Guarantee the legal right to access early childhood care services for all children at risk (health, social).” Cluster 2 represents *Enhancement of living conditions* and contains *Access to income, Access to housing* and *Access to basic infrastructure.* In the second cluster, basic needs, such as housing, a healthy living environment and employment for parents, were targeted. An example of a measure from this cluster is “Negotiate with mayors about enabling access to drinking water, heat, garbage collection and so on.” Cluster 3 represents *Quality and accessibility of health care* and contains topics related to *Prenatal care, Perinatal care, Postnatal care, Paediatric care, Reproductive health and Field health care.* An example of a measure from Cluster 3 is “Implement a system of field paediatric and nursing care.” Cluster 4 represents *Community interventions focused on the transfer of cultural capital* and contains answers to the questions *Who should be educated, By whom, Where should this education take place, How should the education occur* and *What should be the content of the education?* An example of a measure from this cluster is “To support the establishment of maternity centres with a library, toys, sports opportunities for future mothers but also for mothers with children.”

**Fig. 2:**
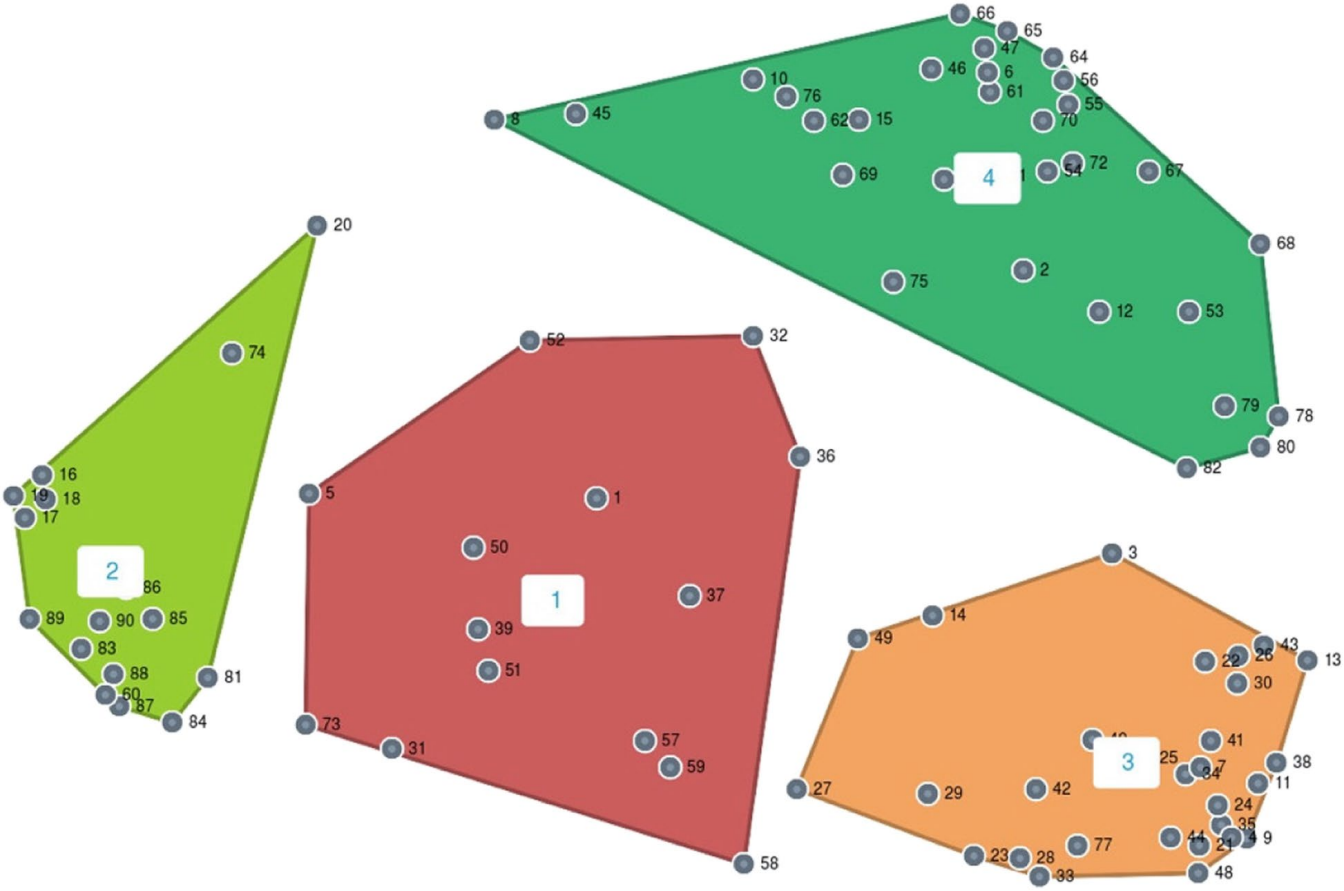
Cluster point map – final 4-cluster solution regarding the proposed measures. Note: Each point represents a measure suggested by participants. The distance between the points indicates how often participants sorted particular measures into the same group (a smaller distance from the centre of a cluster means that more participants placed those particular measures into the same group). The size of the cluster indicates the degree to which its various contributing measures are related

The stress index was 0.1916, which suggests a strong fit between the cluster map and the data, as the value should range from 0.10 to 0.35, with lower values indicating a better fit [20]. The final cluster solution is shown in Fig. 2. All proposed measures and their groups can be seen in the 15.

### Priority measures based on urgency and feasibility

**Fig. 3:**
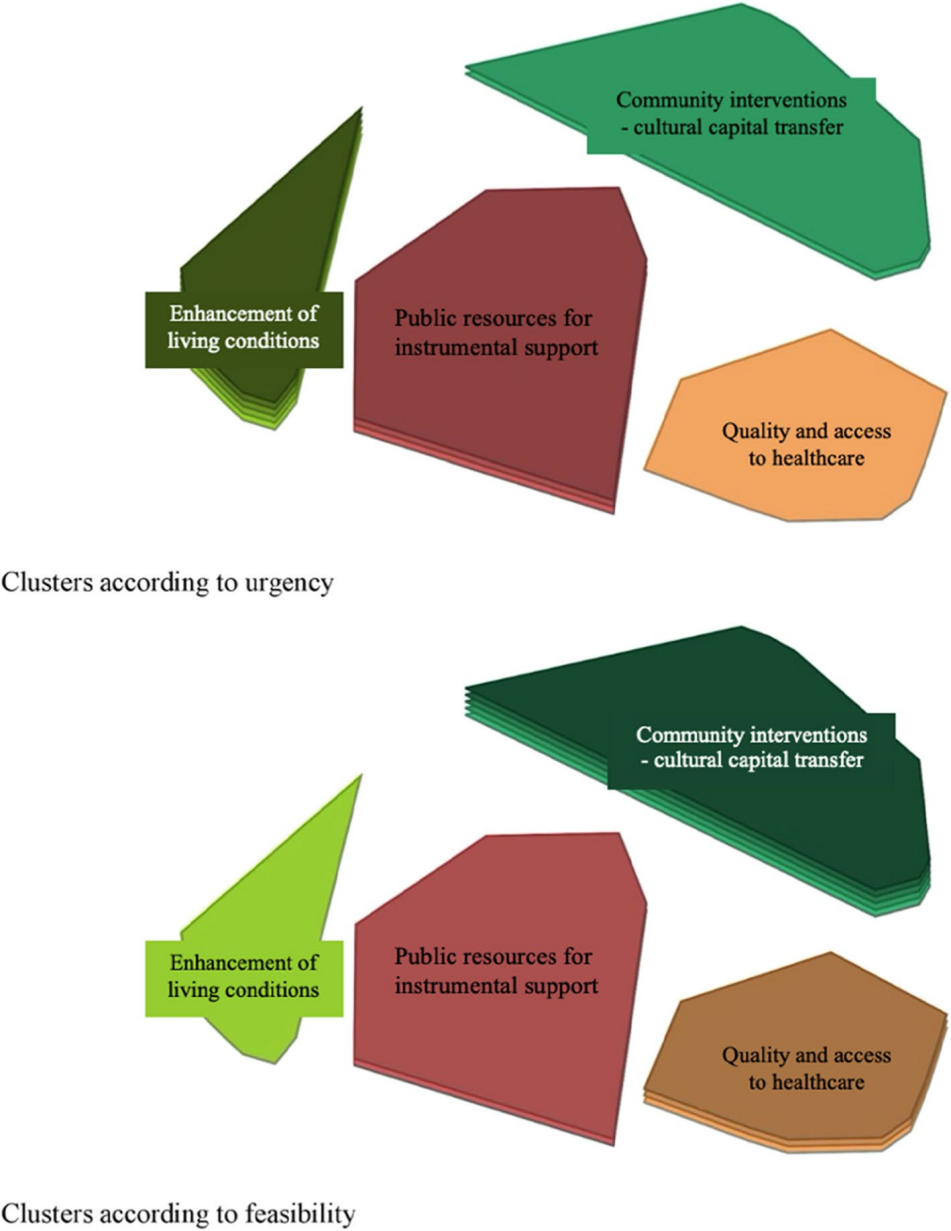
Cluster rating maps regarding urgency and feasibility of the proposed measures. Note: More layers indicate more urgency and feasibility, respectively. The clusters with fewer layers were rated as less urgent (upper map) or less feasible (lower map). The clusters with more layers were rated as more urgent (upper map) or more feasible (lower map)

Regarding *urgency and feasibility of the clusters*, participants considered Cluster 2, i.e. *Enhancement of living conditions* as the most urgent. Cluster 3, i.e. *Quality and accessibility of health care* was considered to be the least urgent. In terms of feasibility, the results were the opposite. Cluster 2, which was considered to be the most urgent cluster, was rated as the least feasible one. Cluster 4 had the highest feasibility, i.e. Community interventions focused on the transfer of cultural capital, despite being rated as one of the least urgent. The urgency and feasibility of the various clusters as rated by participants are shown in Fig. 3.

**Fig. 4:**
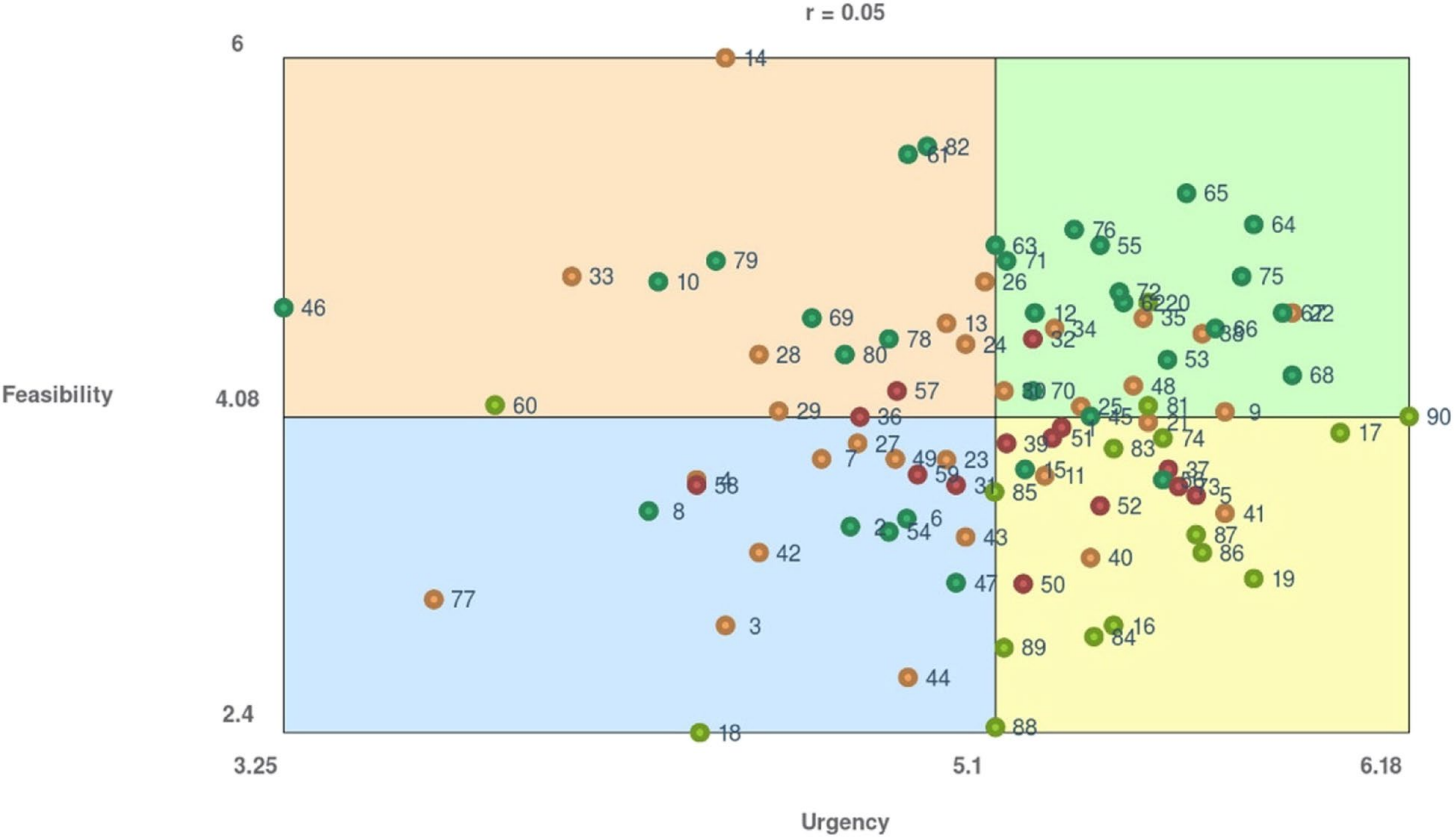
Urgency and feasibility of the proposed measures combined into a “Go-Zone map”. Note: The priority measures rated as the most urgent and most feasible are placed in the green sector in the upper right corner. The colour of a point represents the cluster to which the measure belongs: red—Cluster 1, lime—Cluster 2, orange—Cluster 3, dark green—Cluster 4

Regarding *urgency and feasibility of separate measures,* 27 of the 90 proposed measures were rated as highly urgent and highly feasible, i.e., according to the respondents they should be implemented with a priority in order to equalize the chances of Roma children having healthy development. The identified priority measures according to urgency and feasibility are shown in the Go-Zone map (Fig. 4). All four clusters were represented in these separate measures.

Most priority measures belong to Cluster 4 (*Community interventions focused on the transfer of cultural capital*). Only one of the priority measures belongs to Cluster 1 (*Public resources for instrumental support*). Participants considered the items *“Education and raising awareness of adolescents about reproductive health in schools in cooperation with Roma health mediators”* (item 64) and *“Education and raising awareness of adolescents about reproductive health within community centres”* (item 65) to be the most urgent and feasible. In contrast, the least urgent and feasible was to *“Build a wide network of breast milk banks”* (item 77).

## Discussion

We assessed the perceptions of professionals from different fields working with marginalized Roma communities and identified 90 measures they considered to be needed for healthy early childhood development in children from marginalized Roma communities. These measures were grouped into four clusters, and rated by urgency and feasibility as perceived by participants. Participants identified 27 measures as having priority, i.e., being highly urgent and feasible.

We identified four clusters of measures which mostly align with the sectoral objectives of the EU Roma strategic framework for equality, inclusion and participation [15]. The objectives of this framework regard Housing, Employment, Education, and Health. In the clusters that we identified, Housing and Employment are combined into *Enhancement of living conditions*, Education is the main topic of *Community interventions focused on the transfer of cultural capital*, and issues of Health are addressed within *Quality and accessibility of health care*. Compared to the framework objectives, we identified one additional cluster, i.e., *Public resources for instrumental support,* which includes measures overarching the other three clusters from a policy perspective, enabling access to and strengthening the capacities of the system of care. The grouping of the overarching measures into a separate cluster and not integrated into respective clusters may be interpreted as the participants perceiving the current lack of intersectoral strategies and collaboration in Slovakia as a barrier to the implementation of measures included in other clusters. Slovakia lacks an integrated system of education and care for children aged 0–6 [34]. Such an overarching objective may also add to the EU framework.

Participants perceived the cluster concerning *Enhancement of living conditions* as the most urgent, which aligns with the materialist framework of how social determinants shape health and health outcomes [7]. Within the materialist framework, living conditions are reflected in three key mechanisms influencing health and health outcomes: (1) experience of the material living conditions, (2) psychosocial stress caused by these conditions, and (3) adoption of health-threatening or health-supporting behaviours [7]. The particular measures in the mentioned cluster address living conditions, housing and employment of parents. Their importance is supported by research showing that housing instability and food insecurity do indeed negatively affect health and development in early childhood, with long-term effects in later life [10, 30, 45]. The proposed measures in this cluster thus require action from ministries, such as the Ministry of Labour, Social Affairs and Family, and from municipalities, with some of them also requiring inter-sectoral cooperation.

The cluster concerning *Quality and accessibility of health care* was considered to be the least urgent compared to the other clusters. This seems to contrast the relatively poor quality and accessibility of health care in Slovakia, which are among the lowest of all countries of European Union [16]. Moreover, Roma in Slovakia face significant barriers in access to health care [4, 5]. Hence, an explanation for the low urgency of this cluster may simply be that other clusters contained more urgent issues, or that the interviewed professionals perceive not to need additional policy measures, as they can address this issue themselves. This definitely requires further study.

The cluster of measures regarding *community interventions focused on the transfer of cultural capital* was rated as the most feasible, reflecting the widespread use of community interventions in marginalized communities in Slovakia and across Europe [1]. Currently, several early childhood education and care (ECEC) projects are being realized by NGOs in Slovakia and have been successful on a local level but would need to be scaled up to the national level [55]. Some of the interventions and measures proposed in this cluster may be considered to be an extension of services provided on a community level (such as *Project OMAMA, People in Need Slovakia, Healthy Communities)* [1]. An explanation may be that participants viewed these measures as highly feasible since such interventions already exist, have been shown to be effective and have received public approval. Evidently, this supports further research and development to implement adequate Roma-centred community interventions.

The cluster concerning *enhancement of living conditions* was considered as the least feasible despite being rated as the most urgent, which aligns with the fact that improving the living conditions of marginalized Roma communities in Slovakia has been identified by the EU as a main area requiring EU funding. The spending rate of these EU funds has remained low, possibly due to the resistance of municipalities to use the corresponding schemes [14]. The lack of political will to enhance the living conditions of Roma [42] reflects the public discourse related to poor people’s responsibility for bettering their own living conditions. In this discourse, Roma are labelled as less deserving of public support and consciously abusing the social system [12]. This suggests our participants do not see the political will of municipalities to allocate funds and services for marginalized Roma and to enhance their living conditions. As the measures in this cluster align with the rights guaranteed by Convention on the Rights of the Child [51], which Slovakia signed in 1991, reluctance to implement such measures may be considered a violation of children’s rights.

We also found a significant discrepancy between urgency and feasibility in the cluster related to community interventions focused on the transfer of cultural capital, which was rated as the most feasible but also as one of the least urgent. One of the explanations for the low urgency of this cluster could be that many of the proposed measures already exist and just need to be scaled up. The low appreciation for urgency may also be due to the fact that this cluster contained measures that transfer the responsibility for healthy development onto the parents, rather than making it a public policy issue. Moreover, some of the proposed measures can be viewed as paternalistic, as they do not account for participation and involvement of the Roma community in the design or content of the proposed interventions. Paternalistic measures fail to foster autonomy in the recipient, and therefore such measures replicate the power relations that have contributed to the need for these services in the first place [6]. Participatory measures, on the other hand, promote physical, mental and social health and reduce inequities in health [18]. The general population views Roma as unchangeable and incapable of making wise choices [36]. As a consequence, Roma as a target of policies are perceived as needing directive and authoritarian means and as not capable of participating in enhancing their own conditions. Since many of the measures proposed in this cluster already exist to some extent, participants could feel that the measures in this cluster are not as urgent as those in other clusters.

Within the priority measures, a variety of measures belonging to each of the clusters were included. Measures with the highest urgency and feasibility targeted planning parenthood, on the one hand, and scaling up existing projects on the other hand (See measures 65, 64, 67, 68, 22, 75, and 90 in the 15). The first five of the above-listed measures focus on planning parenthood, in particular on education and awareness about reproductive health, readiness for future parenthood, prenatal development, and the availability of contraception. Roma women get pregnant for the first time earlier than non-Roma women and have a higher number of pregnancies per woman [50]. Unintended pregnancy leads to health-compromising behaviours in pregnancy [32], increases the risk of low birthweight [21] and can lead to negative outcomes for child health and development [11]. Presently, education on sexual, relational and reproductive health, that would meet the international human rights standards, is not available in Slovakia [33]. The high priority given to these measures by our participants suggests that participants view the issues of unwanted and mistimed pregnancies as pressing and the solutions as attainable. Other measures rated as the most urgent and feasible focused on scaling up already existing successful small-scale projects regarding early childhood interventions (Project OMAMA [1];) and access to housing via microloans for self-help construction [38]. These regard examples of good practices in enhancing the living conditions of marginalized Roma [3]. The high priority of these measures reflects the fact that our participants believe in the feasibility of interventions generally viewed as effective and successful.

### Strengths and limitations

The main strength of this study regards the size and quality of the sample, because of its variety, including the viewpoints of diverse stakeholders from practice, research and policy. Stakeholders proposed a set of relevant and acceptable measures, which may help reduce inequalities in the early childhood of children from marginalized Roma communities. Our study also has some limitations, the first being that we lost 14 out the initial 54 participants in the sorting-rating phase of the study. However, this is in accordance with the GCM methodology, which takes into account some losses of participants between the brainstorming and sorting-rating step without bias being likely [24]. Second, the GCM methodology may be prone to social desirability. We reduced the likelihood of this bias by anonymizing the brainstorming phase, which also helped to reduce the potential effects of power relations between participants. Third, the GCM methodology might be prone to subjectivity, typical for qualitative methods, as researchers interact with the data generated by the participants whose selection was purposive. However, we tried to eliminate this as much as possible by using a participatory approach and discussing each step of our study with the participants, implying that in all steps decisions were made by several people jointly. Finally, the interpretations and implications of our finding should be taken with caution, as they were not discussed with representatives of the target population, showing a need for confirmation in future research.

### Implications

Our finding that measures on living conditions are rated as most urgent but least feasible show that this topic evidently deserves further attention regarding the development of measures and their evaluation. The availability of EU funding could facilitate such actions, but evidently the various barriers for use of these funds require further study. Implementing an international monitoring protocol that would collect data both on compliance with the CRC as well as on the developmental outcomes was previously proposed [22]. In the case of violation of children’s rights, the United Nations could utilize legal and political channels to make claims of rights violation.

We identified four clusters and a number of measures that should have priority and which require further interpretation jointly with the target population. The target population could also complete the sorting-rating phase with the same set of proposed measures. Alternatively, this study could be replicated with a different set of stakeholders.

Our findings imply that several policy and practice measures are needed in particular to improve the healthy early childhood development of children living in marginalized Roma communities. The implementation of such measures requires a further involvement of appropriate stakeholders. The participants further identified a high need for coordination and cooperation in addressing the inequalities in early childhood, and hence policymakers should make an effort to approach this matter from a complex perspective. Since Slovakia lacks an integrated system of education and care for children aged 0–6 [34], the creation of such a system is highly needed. Many proposed measures highlight the need for participation. Currently, marginalized Roma hardly participate in policymaking and self-governance [23] and should thus be invited to participate in formulating needs and creating and running programmes aimed at helping Roma. Moreover, our findings show barriers in access to and the capacity of services that should be targeted.

The individual measures that were given the highest priority by the participants highlight the need to address the inability of young Roma to plan their parenthood, so access to methods of contraception should be ensured, as well as access to quality sexual education. Participants gave high priority to measures that would only scale up already existing interventions [1], which means that measures should build upon existing good-practice interventions and programmes.

The prepared summary report of this study in Slovak language will be disseminated among the participants as well as relevant policy makers, to open the discussion about proposed measures and to push for policy change even in those measures, that the participants viewed as not feasible.

## Conclusion

Participants proposed a set of relevant measures that would help equalize the chances of children from marginalized Roma communities for healthy early development, which are in line with European strategic framework for equality, inclusion and participation [15] and which reflect the most pressing issues. Participants viewed the enhancement of living conditions as the most urgent and community interventions focused on the transfer of cultural capital as the most feasible. Discrepancies between urgency and feasibility show significant barriers in the implementation of the proposed measures; they reflect the public discourse and the lack of political will to address the problems of marginalized Roma. The most urgent and feasible measures regarded planning parenthood or were a scaling up of already existing programmes. The proposed need for a supra-sectoral strategy as a separate measure and the variability of proposed measures confirmed that reducing inequalities in early childhood development is a complex issue and needs to be addressed through cross-sectoral cooperation and coordinated efforts.

## Data Availability

All data generated or analysed during this study are included in this published article.

## Appendix

*Final cluster solution (The number of measures as indicated on the cluster map and the go-zone map can be found in the brackets). Measures belonging to the go-zone are in each cluster marked in bold.*

Cluster 1 – Public resources for instrumental support.

1. Establish the institute of Roma assistants (at least available by telephone) and creating a network/database of such assistants who can be contacted by any institution where communication is an essential condition for successful intervention—preschools and primary schools, first contact clinics, foster home facilities, asylum facilities, municipal and state police, etc. (1).
2. The state should increase the overall allocation of funds to education, financially support the creation and operation of programmes for children younger than 3 years. (5).
3. Increase the number of controls by the social protection of children, mapping the care of children in the period before and after childbirth, supervision of families with children, regular family visits. (31).
4. **Strengthening the capacities of trained social workers in the field who know the customs, culture and mentality of marginalized Roma communities. (32).**
5. Introduce the positions of parental assistants/trustees into the environment of facilities for children aged 0–6 years, who would help bridge the needs/barriers of the family with the content of these institutions. (36).
6. Ensure state-paid Roma field assistants in each Marginalized Roma Community (MRC), so that a child is caught as soon as he/she arrives from the maternity ward and subsequently monitored and so that parents are monitored to see if the child has health insurance coverage, a birth certificate, a district paediatrician and preventive examinations. Implement family supervision. (37).
7. Create a grant programme to support NGOs providing early childhood care in the field. (39).
8. Financially and institutionally support Early Intervention Centres and create a regionally accessible network of centres that are able to provide interventions in the field and specifically in MRC (network expansion, strengthening human resources, technical and material security and strategies for further development). (50).
9. Guarantee the legal right to access early childhood care services for all at-risk children (health, social). (51).
10. Develop a supra-ministerial strategy for early childhood education and care as a cross-sectoral and multidisciplinary system (in reconciling work and family life, health, social services and education), including standards for early childhood care provision, a monitoring system of quality and a funding system. (52).
11. Starter kits of basic equipment for a new-born child instead of a financial benefit at the birth of a child conditioned by gynaecological examinations during pregnancy. (57).
12. Introduction of dairy snacks and fruit and vegetable rations for children younger than 3 years through municipalities. (58).
13. Meal allowance for pregnant women and mothers of children younger than 3 years in the form of coupons for milk, fruit, vegetables, breast milk substitutes and vitamins (following the example of the United Kingdom). (59).
14. Ensure the continuity of financing early care programmes from the state budget within the social policy of the state, e.g. through municipal enterprises or the Office of the Government Plenipotentiary for the Roma Community. (73).

Cluster 2 – Enhancement of living conditions.

1. Improve access to income for the Roma population (by introducing sheltered employment programmes in companies, training on the part of employers, creating tax benefits and incentive bonuses). (16).
2. Increase Roma employment through social enterprises. (17).
3. The introduction of a “mandatory quota” for the employment of Roma, similar to that for the disadvantaged, and in the case of non-compliance, require alternative benefits in the form of financial support for NGO programmes that address the issue and replace the state. (18).
4. Create as many job opportunities as possible for parents so that they can raise their standard of living. (19).
5. **Implement media campaigns aimed at changing the attitudes of the majority towards MRC, sensitizing public opinion, supporting critical thinking, fighting against hoaxes and misinformation. (20).**
6. Payment of parental benefits at weekly intervals (not once a month). (60).
7. Employ women from communities that provide early childhood care on full-time contracts. (74).
8. **Enable access to water and hygiene – through community centres or local authorities – by paying a “fee” for water, etc. (81).**
9. Negotiate with mayors about enabling access to drinking water, heat, garbage collection and so on. (83).
10. Special legislation on access to drinking water and legislative removal of obstacles to the management of infrastructure for water networks, including the law on the removal of all obstacles to the supply of drinking water to all households, regardless of whether it is a legal or illegal building. (84).
11. Ensure the cooperation of relevant actors (municipality, self-governing region, private sector) in waste collection. (85).
12. Ensure the cooperation of relevant actors (municipality, self-governing region, private sector) in building infrastructure (water, sewerage, utilities, roads and sidewalks). (86).
13. To support the motivation of municipalities to address the issue of housing in MRC from the government level. (87).
14. Ensure the legalization of building parcels. (88).
15. Ensure the construction of social housing. (89).
16. **Improve access to housing in the form of microloans for the self-help construction of new flats/houses. (90).**

Cluster 3 – Quality and availability of health care.

1. Provide mothers after delivery with support and help with taking care of older children etc., so that they can regenerate and start breastfeeding successfully. (3).
2. Introduce mandatory counselling centres for women in gynaecological clinics. Establish maternal blood alcohol measurements. In the case of a positive result, impose sanctions. This measure prevents high maternal alcohol consumption and also the possible occurrence of FAS (foetal alcohol syndrome). (4).
3. Create a programme to eliminate childhood anaemia along the lines of WHO programmes (early detection and management, nutrition promotion and supplementation, especially in low birth weight children and pregnant women and mothers with low BMI, routine vaccination and deworming, parental counselling). (7).
4. **Ensure real health care in paediatric outpatient clinics. Many children from MRC are not monitored by a paediatrician and parents solve their health problems through the emergency room. Prevention is not available to them. (9).**
5. Provide multidisciplinary support immediately after childbirth (nurse together with a doctor, social worker, field worker). (11).
6. Carry out an examination of psychomotor development with the participation of Roma health mediators or with the workers providing early childhood care. (13).
7. Translate a questionnaire aimed at examining psychomotor development into the language of ethnic minorities. (14).
8. Improve the availability of prenatal care by requiring the corresponding GP to be obliged to take every woman from his or her district into care. Provide the assistance of field workers in this process, so that the woman cannot be rejected. (21).
9. **Improve the availability of contraception. (22).**
10. An application for a certain form of contraception (the client chooses the type herself) should be part of the application for a social benefit in material need. The chosen form of contraception should not be free but for a certain symbolic fee appropriate to the type of contraception. (23).
11. Introduce a mandatory code of equal and fair access from staff towards MRC in health care. (24).
12. **A Roma health mediator in each hospital as a connecting bridge between MRC and the staff. (25).**
13. Educate doctors and hospital staff on minority rights and communication to eliminate discriminatory behaviour. (26).
14. Adopt legislation to prevent obstetric violence. (27).
15. Carry out research on obstetric violence against Roma women and violations of the human rights of Roma women and children during childbirth and hospitalization to eliminate traumatizing and discriminatory practices. (28).
16. End various stigmatizing practices in paediatric wards by law – for example, shaving the heads of Roma children who have lice etc. (29).
17. **More thorough work with mothers in prenatal clinics, e.g. through nurses. (30).**
18. Enable women to leave the maternity ward with their baby earlier, if the baby’s health condition allows it without stigmatization and financial barriers. (33).
19. **Provide assistants in maternity wards who would provide information on the new-born care, lactation counselling, lifestyle. (34).**
20. **Work with paediatricians, support the implementation of screenings, referrals to specialists in case of suspected developmental delays. (35).**
21. **Encourage the presence of health mediators in MRC and their cooperation with paediatricians to train mothers in the correct distinction of the need to visit a GP or emergency services, the correct administration and dosing of medicines, first aid for life-threatening conditions and the needs of the child. (38).**
22. Implement a system of field paediatric and nursing care. (40).
23. Implement a system of field nurses who would visit mothers in their homes during the postpartum period, teach and help with individual aspects of new-born care. (41).
24. Set up a clinic at a municipal office or community centre for a specified time of the week (e.g. an outpatient clinic for children and adolescents, a gynaecological outpatient clinic). (42).
25. Daily visitations of mothers in the postpartum period by a Roma health mediator, who will mentor the mother during this period, teaching her everything she needs and supporting her. (43).
26. Create mobile gynaecological, paediatric and dental clinics and thus ensure compliance with examinations, vaccinations, necessary examinations in remote MRCs. (44).
27. **Ensure access for Roma children to better preventive care that filters out congenital problems that can be addressed immediately. (48).**
28. Introduce a mobile service, or a field early intervention service, that will focus not only on health disadvantages but also on social disadvantages and prevention. (49).
29. Build a wide network of breast milk banks. (77).

Cluster 4 – Community interventions focused on the transfer of cultural capital.

1. Establish and support the operation of field special educators for children younger than 3 years, operating under the Centres of pedagogical-psychological counselling and prevention. (2).
2. Education of Roma women. Teach them self-sufficiency, independence from their partner. Teach them a craft so that they can integrate into the work process after parental leave. During maternal leave and parental leave, they could undergo some personality training. (6).
3. Build community gardens. Lead families to grow crops, ensuring vegetables and fruits for themselves and their children. (8).
4. Ensure the lending of educational materials and resources to households with children in MRC. (10).
5. **Create training programmes for professionals providing early childhood care. (12).**
6. Create preparatory classes for children who are younger (they could be created, for example, at the level of community centres), where trained workers would work with children and prepare them for entering preschool. (15).
7. **Teach MRC members to manage money, increase their financial literacy and also teach them self-sufficiency. (45).**
8. Teach parents how to make simple toys. (46).
9. Educate parents in the Slovak language, lead them to talk to children, to read. (47).
10. **Individual support, education and raising awareness in the households of pregnant women in the field of caring for their health and the health of their unborn child (healthy lifestyle, substance abuse) using experiential methods. (53).**
11. Introduction of compulsory family education (instruction on a child’s psychomotor development, attachment and emotional deprivation, but also obligations arising from legislation) to all families (including non-Roma) who are expecting their first addition. (54).
12. **Educational meetings – discussions, lectures, interesting activities for pregnant women and future fathers from MRC (e.g. in cooperation with Roma health mediators) with the active participation of a paediatrician and gynaecologist focused on prenatal care for women, the importance of postpartum care, new-born care, psychomotor development, proper nutrition, hygiene. Practical demonstrations at group meetings. (55).**
13. Mandatory educational activities (on child development and care, responsible parenting...) for all underage mothers, regardless of ethnicity during pregnancy and after childbirth. (56).
14. Educate parents on how to play with children, how to draw with crayons and chalks, in the community centre. (61).
15. **Expand the scope of activities in the community centres so that mothers with children can spend time in them as an alternative to pre-school education. (62).**
16. Include the creation of clubs for mothers with children under 3 years of age in the activities of community centres as a mandatory activity of the community centre. (63).
17. 17. **Education and raising awareness of adolescents about reproductive health in schools in cooperation with Roma health mediators. (64).**
18. **Education and raising awareness of adolescents about reproductive health within community centres. (65).**
19. **Develop education, mentoring and incentive programmes for young people in primary schools in order to show them an alternative to early parenthood. (66).**
20. **A comprehensive education in the field of readiness for future parenthood and on healthy prenatal development. (67).**
21. **A comprehensive education in the field of reproductive health providing prevention of unwanted pregnancy, but also about readiness for future parenthood and healthy prenatal development. (68).**
22. To support the establishment of maternity centres with a library, toys, sports opportunities for future mothers but also mothers with children. (69).
23. **Create educational programmes for parents, including fathers, focused on early childhood care (including, e.g., support for parental competencies, care, development, nutrition, development of children without costly educational aids, experiential learning, emotional attachment, elements of Montessori education, reading and singing with children, playing, drawing, making simple toys, etc.). (70).**
24. **Support parent clubs, where parents meet either on a self-help basis and support each other, exchange experiences, or based on support groups with experts, such as paediatricians, lactation counsellors, early intervention centre experts. (71).**
25. **Support and education of parents about parenting skills, care and child nutrition by field workers in the natural home environment. (72).**
26. **Support projects where women from communities come into families and work with children. (75).**
27. **Involve people from the community in the implementation of programmes for parents, including their training and preparation. (76).**
28. Implement individual prenatal courses on breastfeeding in households of pregnant women and new mothers in MRC. (78).
29. Raise awareness, support and promote breastfeeding by creating support groups for breastfeeding mothers. (79).
30. Train lactation counsellors from among the mothers from MRC who have experience with breastfeeding to provide qualified lactation counselling in MRC. (80).
31. Raise awareness on the sanitation of containers used for water as prevention of intestinal problems. (82).
